# Supplementary material for: Pyruvate Dehydrogenase Kinase 1 inhibition mediated oxidative phosphorylation enhancement in cartilage promotes osteoarthritis progression
Source: BMC Musculoskelet Disord. 2023 Jul 20;24:597. doi: 10.1186/s12891-023-06585-6 (PMC10357736; doi:10.1186/s12891-023-06585-6)
Supplement: Supplementary file 1 — Additional file 1. [file 12891_2023_6585_MOESM1_ESM.zip › MF.Gsea.1653627316242/GOMF_TRANSCRIPTION_COREGULATOR_BINDING.html]

Details for gene set GOMF\_TRANSCRIPTION\_COREGULATOR\_BINDING[GSEA]

|  || Dataset | 55457\_collapsed\_to\_symbols.cls.cls #OA\_versus\_normal.cls.cls #OA\_versus\_normal\_repos |
| Phenotype | cls.cls#OA\_versus\_normal\_repos |
| Upregulated in class | normal |
| GeneSet | GOMF\_TRANSCRIPTION\_COREGULATOR\_BINDING |
| Enrichment Score (ES) | -0.4371382 |
| Normalized Enrichment Score (NES) | -1.8896377 |
| Nominal p-value | 0.0 |
| FDR q-value | 0.040665496 |
| FWER p-Value | 0.232 |
Table: GSEA Results Summary

  

Fig 1: Enrichment plot: GOMF\_TRANSCRIPTION\_COREGULATOR\_BINDING      
 Profile of the Running ES Score & Positions of GeneSet Members on the Rank Ordered List

  

| SYMBOL | TITLE | RANK IN GENE LIST | RANK METRIC SCORE | RUNNING ES | CORE ENRICHMENT || 1 | PAX6 | paired box 6 [Source:HGNC Symbol;Acc:HGNC:8620] | 304 | 0.746 | -0.0015 | No |
| 2 | EED | embryonic ectoderm development [Source:HGNC Symbol;Acc:HGNC:3188] | 454 | 0.680 | 0.0071 | No |
| 3 | ELOB | elongin B [Source:HGNC Symbol;Acc:HGNC:11619] | 933 | 0.531 | -0.0144 | No |
| 4 | ESR1 | estrogen receptor 1 [Source:HGNC Symbol;Acc:HGNC:3467] | 984 | 0.522 | -0.0028 | No |
| 5 | HDAC3 | histone deacetylase 3 [Source:HGNC Symbol;Acc:HGNC:4854] | 1325 | 0.454 | -0.0158 | No |
| 6 | MED6 | mediator complex subunit 6 [Source:HGNC Symbol;Acc:HGNC:19970] | 1744 | 0.393 | -0.0368 | No |
| 7 | USP11 | ubiquitin specific peptidase 11 [Source:HGNC Symbol;Acc:HGNC:12609] | 1929 | 0.369 | -0.0402 | No |
| 8 | SIX1 | SIX homeobox 1 [Source:HGNC Symbol;Acc:HGNC:10887] | 2011 | 0.360 | -0.0358 | No |
| 9 | NFE2L2 | "nuclear factor, erythroid 2 like 2 [Source:HGNC Symbol;Acc:HGNC:7782]" | 2034 | 0.357 | -0.0269 | No |
| 10 | LMO2 | LIM domain only 2 [Source:HGNC Symbol;Acc:HGNC:6642] | 2122 | 0.347 | -0.0233 | No |
| 11 | CTCF | CCCTC-binding factor [Source:HGNC Symbol;Acc:HGNC:13723] | 2183 | 0.340 | -0.0179 | No |
| 12 | PER3 | period circadian regulator 3 [Source:HGNC Symbol;Acc:HGNC:8847] | 2575 | 0.293 | -0.0397 | No |
| 13 | NFATC1 | nuclear factor of activated T cells 1 [Source:HGNC Symbol;Acc:HGNC:7775] | 3027 | 0.247 | -0.0676 | No |
| 14 | UBE2I | ubiquitin conjugating enzyme E2 I [Source:HGNC Symbol;Acc:HGNC:12485] | 3424 | 0.209 | -0.0923 | No |
| 15 | PBX1 | PBX homeobox 1 [Source:HGNC Symbol;Acc:HGNC:8632] | 3887 | 0.166 | -0.1234 | No |
| 16 | RELA | "RELA proto-oncogene, NF-kB subunit [Source:HGNC Symbol;Acc:HGNC:9955]" | 3942 | 0.161 | -0.1228 | No |
| 17 | SMAD3 | SMAD family member 3 [Source:HGNC Symbol;Acc:HGNC:6769] | 4061 | 0.150 | -0.1276 | No |
| 18 | HDAC5 | histone deacetylase 5 [Source:HGNC Symbol;Acc:HGNC:14068] | 4336 | 0.125 | -0.1453 | No |
| 19 | STAT1 | signal transducer and activator of transcription 1 [Source:HGNC Symbol;Acc:HGNC:11362] | 4495 | 0.113 | -0.1542 | No |
| 20 | SREBF1 | sterol regulatory element binding transcription factor 1 [Source:HGNC Symbol;Acc:HGNC:11289] | 4729 | 0.093 | -0.1697 | No |
| 21 | SMARCD3 | "SWI/SNF related, matrix associated, actin dependent regulator of chromatin, subfamily d, member 3 [Source:HGNC Symbol;Acc:HGNC:11108]" | 5036 | 0.070 | -0.1915 | No |
| 22 | CTBP2 | C-terminal binding protein 2 [Source:HGNC Symbol;Acc:HGNC:2495] | 5066 | 0.067 | -0.1917 | No |
| 23 | EHMT2 | euchromatic histone lysine methyltransferase 2 [Source:HGNC Symbol;Acc:HGNC:14129] | 5507 | 0.032 | -0.2252 | No |
| 24 | GATA6 | GATA binding protein 6 [Source:HGNC Symbol;Acc:HGNC:4174] | 5537 | 0.030 | -0.2265 | No |
| 25 | CNOT2 | CCR4-NOT transcription complex subunit 2 [Source:HGNC Symbol;Acc:HGNC:7878] | 5740 | 0.012 | -0.2419 | No |
| 26 | TERT | telomerase reverse transcriptase [Source:HGNC Symbol;Acc:HGNC:11730] | 5773 | 0.010 | -0.2441 | No |
| 27 | RUNX3 | RUNX family transcription factor 3 [Source:HGNC Symbol;Acc:HGNC:10473] | 5860 | 0.004 | -0.2507 | No |
| 28 | MTF2 | metal response element binding transcription factor 2 [Source:HGNC Symbol;Acc:HGNC:29535] | 5996 | -0.004 | -0.2612 | No |
| 29 | PPARG | peroxisome proliferator activated receptor gamma [Source:HGNC Symbol;Acc:HGNC:9236] | 6006 | -0.005 | -0.2617 | No |
| 30 | SIX3 | SIX homeobox 3 [Source:HGNC Symbol;Acc:HGNC:10889] | 6356 | -0.032 | -0.2880 | No |
| 31 | THAP7 | THAP domain containing 7 [Source:HGNC Symbol;Acc:HGNC:23190] | 6412 | -0.036 | -0.2912 | No |
| 32 | ELOC | elongin C [Source:HGNC Symbol;Acc:HGNC:11617] | 6556 | -0.046 | -0.3010 | No |
| 33 | EZH2 | enhancer of zeste 2 polycomb repressive complex 2 subunit [Source:HGNC Symbol;Acc:HGNC:3527] | 6562 | -0.047 | -0.3000 | No |
| 34 | HDGF | heparin binding growth factor [Source:HGNC Symbol;Acc:HGNC:4856] | 6924 | -0.074 | -0.3260 | No |
| 35 | NR1D1 | nuclear receptor subfamily 1 group D member 1 [Source:HGNC Symbol;Acc:HGNC:7962] | 6974 | -0.078 | -0.3275 | No |
| 36 | RXRA | retinoid X receptor alpha [Source:HGNC Symbol;Acc:HGNC:10477] | 7381 | -0.109 | -0.3560 | No |
| 37 | PHF1 | PHD finger protein 1 [Source:HGNC Symbol;Acc:HGNC:8919] | 7429 | -0.113 | -0.3563 | No |
| 38 | LEF1 | lymphoid enhancer binding factor 1 [Source:HGNC Symbol;Acc:HGNC:6551] | 7467 | -0.116 | -0.3558 | No |
| 39 | PER2 | period circadian regulator 2 [Source:HGNC Symbol;Acc:HGNC:8846] | 7663 | -0.132 | -0.3671 | No |
| 40 | HDAC6 | histone deacetylase 6 [Source:HGNC Symbol;Acc:HGNC:14064] | 7833 | -0.143 | -0.3760 | No |
| 41 | HDAC1 | histone deacetylase 1 [Source:HGNC Symbol;Acc:HGNC:4852] | 7911 | -0.148 | -0.3776 | No |
| 42 | CCNT2 | cyclin T2 [Source:HGNC Symbol;Acc:HGNC:1600] | 8192 | -0.170 | -0.3944 | No |
| 43 | HAND2 | heart and neural crest derivatives expressed 2 [Source:HGNC Symbol;Acc:HGNC:4808] | 8211 | -0.172 | -0.3907 | No |
| 44 | AR | androgen receptor [Source:HGNC Symbol;Acc:HGNC:644] | 8217 | -0.173 | -0.3859 | No |
| 45 | NR1H4 | nuclear receptor subfamily 1 group H member 4 [Source:HGNC Symbol;Acc:HGNC:7967] | 8481 | -0.192 | -0.4007 | No |
| 46 | CHD4 | chromodomain helicase DNA binding protein 4 [Source:HGNC Symbol;Acc:HGNC:1919] | 8917 | -0.226 | -0.4280 | No |
| 47 | SMAD4 | SMAD family member 4 [Source:HGNC Symbol;Acc:HGNC:6770] | 9035 | -0.235 | -0.4302 | Yes |
| 48 | PGR | progesterone receptor [Source:HGNC Symbol;Acc:HGNC:8910] | 9048 | -0.235 | -0.4241 | Yes |
| 49 | HAND1 | heart and neural crest derivatives expressed 1 [Source:HGNC Symbol;Acc:HGNC:4807] | 9082 | -0.238 | -0.4196 | Yes |
| 50 | LCOR | ligand dependent nuclear receptor corepressor [Source:HGNC Symbol;Acc:HGNC:29503] | 9227 | -0.250 | -0.4234 | Yes |
| 51 | AHR | aryl hydrocarbon receptor [Source:HGNC Symbol;Acc:HGNC:348] | 9263 | -0.253 | -0.4186 | Yes |
| 52 | ATF7 | activating transcription factor 7 [Source:HGNC Symbol;Acc:HGNC:792] | 9281 | -0.254 | -0.4123 | Yes |
| 53 | CIT | citron rho-interacting serine/threonine kinase [Source:HGNC Symbol;Acc:HGNC:1985] | 9356 | -0.260 | -0.4104 | Yes |
| 54 | ENO1 | enolase 1 [Source:HGNC Symbol;Acc:HGNC:3350] | 9606 | -0.284 | -0.4214 | Yes |
| 55 | ETS1 | "ETS proto-oncogene 1, transcription factor [Source:HGNC Symbol;Acc:HGNC:3488]" | 9795 | -0.301 | -0.4271 | Yes |
| 56 | THRB | thyroid hormone receptor beta [Source:HGNC Symbol;Acc:HGNC:11799] | 9820 | -0.303 | -0.4199 | Yes |
| 57 | BAIAP2 | BAR/IMD domain containing adaptor protein 2 [Source:HGNC Symbol;Acc:HGNC:947] | 9892 | -0.311 | -0.4162 | Yes |
| 58 | HMGA1 | high mobility group AT-hook 1 [Source:HGNC Symbol;Acc:HGNC:5010] | 9966 | -0.318 | -0.4125 | Yes |
| 59 | SMARCA4 | "SWI/SNF related, matrix associated, actin dependent regulator of chromatin, subfamily a, member 4 [Source:HGNC Symbol;Acc:HGNC:11100]" | 10009 | -0.322 | -0.4062 | Yes |
| 60 | SUZ12 | SUZ12 polycomb repressive complex 2 subunit [Source:HGNC Symbol;Acc:HGNC:17101] | 10104 | -0.331 | -0.4037 | Yes |
| 61 | RARA | retinoic acid receptor alpha [Source:HGNC Symbol;Acc:HGNC:9864] | 10141 | -0.335 | -0.3965 | Yes |
| 62 | CTBP1 | C-terminal binding protein 1 [Source:HGNC Symbol;Acc:HGNC:2494] | 10188 | -0.339 | -0.3900 | Yes |
| 63 | ZBTB16 | zinc finger and BTB domain containing 16 [Source:HGNC Symbol;Acc:HGNC:12930] | 10246 | -0.346 | -0.3841 | Yes |
| 64 | NR5A1 | nuclear receptor subfamily 5 group A member 1 [Source:HGNC Symbol;Acc:HGNC:7983] | 10308 | -0.351 | -0.3785 | Yes |
| 65 | VGLL4 | vestigial like family member 4 [Source:HGNC Symbol;Acc:HGNC:28966] | 10338 | -0.354 | -0.3702 | Yes |
| 66 | FOS | "Fos proto-oncogene, AP-1 transcription factor subunit [Source:HGNC Symbol;Acc:HGNC:3796]" | 10640 | -0.387 | -0.3822 | Yes |
| 67 | CREBBP | CREB binding protein [Source:HGNC Symbol;Acc:HGNC:2348] | 10881 | -0.414 | -0.3886 | Yes |
| 68 | GATA1 | GATA binding protein 1 [Source:HGNC Symbol;Acc:HGNC:4170] | 10958 | -0.425 | -0.3818 | Yes |
| 69 | ZBTB17 | zinc finger and BTB domain containing 17 [Source:HGNC Symbol;Acc:HGNC:12936] | 10996 | -0.429 | -0.3720 | Yes |
| 70 | GATA2 | GATA binding protein 2 [Source:HGNC Symbol;Acc:HGNC:4171] | 11058 | -0.439 | -0.3637 | Yes |
| 71 | CDK9 | cyclin dependent kinase 9 [Source:HGNC Symbol;Acc:HGNC:1780] | 11071 | -0.441 | -0.3515 | Yes |
| 72 | PPARA | peroxisome proliferator activated receptor alpha [Source:HGNC Symbol;Acc:HGNC:9232] | 11110 | -0.445 | -0.3412 | Yes |
| 73 | CBX3 | chromobox 3 [Source:HGNC Symbol;Acc:HGNC:1553] | 11113 | -0.445 | -0.3281 | Yes |
| 74 | MED1 | mediator complex subunit 1 [Source:HGNC Symbol;Acc:HGNC:9234] | 11146 | -0.450 | -0.3172 | Yes |
| 75 | WIZ | WIZ zinc finger [Source:HGNC Symbol;Acc:HGNC:30917] | 11226 | -0.461 | -0.3097 | Yes |
| 76 | PPARD | peroxisome proliferator activated receptor delta [Source:HGNC Symbol;Acc:HGNC:9235] | 11301 | -0.471 | -0.3014 | Yes |
| 77 | FOXO3 | forkhead box O3 [Source:HGNC Symbol;Acc:HGNC:3821] | 11305 | -0.472 | -0.2876 | Yes |
| 78 | NR5A2 | nuclear receptor subfamily 5 group A member 2 [Source:HGNC Symbol;Acc:HGNC:7984] | 11362 | -0.481 | -0.2777 | Yes |
| 79 | ZBTB7A | zinc finger and BTB domain containing 7A [Source:HGNC Symbol;Acc:HGNC:18078] | 11600 | -0.516 | -0.2808 | Yes |
| 80 | EHMT1 | euchromatic histone lysine methyltransferase 1 [Source:HGNC Symbol;Acc:HGNC:24650] | 11686 | -0.530 | -0.2717 | Yes |
| 81 | TWIST1 | twist family bHLH transcription factor 1 [Source:HGNC Symbol;Acc:HGNC:12428] | 11696 | -0.531 | -0.2566 | Yes |
| 82 | EP300 | E1A binding protein p300 [Source:HGNC Symbol;Acc:HGNC:3373] | 11741 | -0.540 | -0.2440 | Yes |
| 83 | EPAS1 | endothelial PAS domain protein 1 [Source:HGNC Symbol;Acc:HGNC:3374] | 11758 | -0.542 | -0.2291 | Yes |
| 84 | HIF1A | hypoxia inducible factor 1 subunit alpha [Source:HGNC Symbol;Acc:HGNC:4910] | 11833 | -0.556 | -0.2183 | Yes |
| 85 | TFAM | "transcription factor A, mitochondrial [Source:HGNC Symbol;Acc:HGNC:11741]" | 12175 | -0.637 | -0.2260 | Yes |
| 86 | GATA3 | GATA binding protein 3 [Source:HGNC Symbol;Acc:HGNC:4172] | 12276 | -0.670 | -0.2139 | Yes |
| 87 | MED25 | mediator complex subunit 25 [Source:HGNC Symbol;Acc:HGNC:28845] | 12480 | -0.741 | -0.2077 | Yes |
| 88 | PER1 | period circadian regulator 1 [Source:HGNC Symbol;Acc:HGNC:8845] | 12496 | -0.746 | -0.1866 | Yes |
| 89 | SP1 | Sp1 transcription factor [Source:HGNC Symbol;Acc:HGNC:11205] | 12557 | -0.777 | -0.1682 | Yes |
| 90 | RPL23 | ribosomal protein L23 [Source:HGNC Symbol;Acc:HGNC:10316] | 12558 | -0.777 | -0.1450 | Yes |
| 91 | BCL6 | BCL6 transcription repressor [Source:HGNC Symbol;Acc:HGNC:1001] | 12667 | -0.838 | -0.1285 | Yes |
| 92 | HES1 | hes family bHLH transcription factor 1 [Source:HGNC Symbol;Acc:HGNC:5192] | 12676 | -0.849 | -0.1039 | Yes |
| 93 | RORA | RAR related orphan receptor A [Source:HGNC Symbol;Acc:HGNC:10258] | 12696 | -0.862 | -0.0797 | Yes |
| 94 | MYC | "MYC proto-oncogene, bHLH transcription factor [Source:HGNC Symbol;Acc:HGNC:7553]" | 12801 | -0.970 | -0.0590 | Yes |
| 95 | JUND | "JunD proto-oncogene, AP-1 transcription factor subunit [Source:HGNC Symbol;Acc:HGNC:6206]" | 12865 | -1.116 | -0.0307 | Yes |
| 96 | CTNNB1 | catenin beta 1 [Source:HGNC Symbol;Acc:HGNC:2514] | 12866 | -1.119 | 0.0027 | Yes |
Table: GSEA details [plain text format]

  

Fig 2: GOMF\_TRANSCRIPTION\_COREGULATOR\_BINDING      
 Blue-Pink O' Gram in the Space of the Analyzed GeneSet

  

Fig 3: GOMF\_TRANSCRIPTION\_COREGULATOR\_BINDING: Random ES distribution      
 Gene set null distribution of ES for **GOMF\_TRANSCRIPTION\_COREGULATOR\_BINDING**
